# Evaluating a stress management intervention effects on post-ICU syndrome in families: study protocol for a single-center, prospective, parallel-design, randomized controlled trial

**DOI:** 10.3389/fpsyg.2026.1783279

**Published:** 2026-04-13

**Authors:** Qinqin Li, Tingrui Wang, Zhangyi Wang, Jiajia Yin, Yan Liu, Zihan Zhou, Lihua Xia, Gang Lei, Jie Yang, Zhigang Zhang, Li Yao

**Affiliations:** 1 School of Nursing, Guizhou Medical University, Guiyang, Guizhou, China; 2 Gastrointestinal Surgery/ National Key Clinical Construction Specialist General Surgery, Clinical Medical Technology Demonstration Base for Emergency Treatment of Chest Pain in Hunan Province, the Affiliated Hengyang Hospital of Hunan Normal University & Hengyang Central Hospital, Hengyang, China; 3 President’s Office, Hengyang Fifth People’s Hospital, Hengyang, China; 4 Department of Critical Care Medicine, First Hospital of Lanzhou University, Lanzhou, Gansu, China; 5 School of Nursing, Lanzhou University, Lanzhou, Gansu, China; 6 Department of Respiratory and Critical Care Medicine, The Affiliated Hospital of Guizhou Medical University, Guiyang, Guizhou, China

**Keywords:** ICU, post-intensive care syndrome-family, psychological intervention, randomized controlled trial, stress management

## Abstract

**Aim(s):**

The aim of this study is to assess the impact of a stress management intervention on the prevention of Post-Intensive Care Syndrome-Family (PICS-F).

**Design:**

A single-center, prospective, parallel-design, randomized controlled trial (RCT) study.

**Methods:**

This study will recruit 56 family members of ICU patients from a hospital in China. Participants will be randomly assigned to either the experimental group or the control group. The control group will receive standard care, and the experimental group will receive standard care plus a stress management intervention. The primary outcome is a composite score of psychological distress (integrating perceived stress, anxiety, and depression) measured immediately post-intervention. Secondary outcomes include the longitudinal trajectories of individual psychological symptoms (stress, anxiety, depression), symptoms of post-traumatic stress, and sleep quality across multiple follow-ups up to 90 days. Data analysis will be performed utilizing IBM SPSS version 27.0 software.

**Discussion:**

This study protocol describes a RCT aimed at evaluating the effectiveness of the stress management intervention measures in preventing the occurrence of the PICS-F among family members of patients in the intensive care unit.

**Clinical trial registration:**

https://www.chictr.org.cn/bin/project/edit?pid=272710, identifier ChiCTR2500102953.

## 1 Introduction

In the Intensive Care Unit (ICU), the primary objective of the medical staff is to preserve patients’ lives and minimize the incidence of complications, given the critical nature of their conditions (Laux et al., 2023). For the families of patients, medical staff are usually limited to informing the patients of the current status of the disease, treatment measures and progress, and pay less attention to the psychological and emotional support of the families (Gu et al., 2023; Li and Huang, 2020; Mi et al., 2021; Ran, et al., 2023). ICU patients are often in critical condition, with many in a comatose state. The physical and mental health of family members, who serve as primary caregivers and clinical decision-makers, significantly influence the patients’ eventual clinical recovery outcomes (Lin et al., 2020). The high cost of treatment, sudden hospitalization of critically ill relatives, restricted ICU visitation hours, lack of familiarity with the ICU environment, fear of uncertain disease prognosis, and the need to make clinical decisions on behalf of patients—all these factors impose a heavy burden on family members, leading to physical, psychological, and social dysfunction, a condition referred to as Post-Intensive Care Syndrome-Family (PICS-F) (Davidson et al., 2012; Davidson and Harvey, 2016). Among these, psychological disorders encompass stress, anxiety, depression, post-traumatic stress disorder (PTSD), and complex grief, with anxiety and depression being the most prevalent (Hayes et al., 2024).

Research indicates that the prevalence of PICS-F ranges from 6 to 69% within 6 months following the discharge of patients from the ICU (Petrinec and Martin, 2018). 58.1% of family members are likely to experience moderate to severe sleep disturbances, while 57.6% may suffer from significant fatigue (Dayet et al., 2013). In a survey conducted among 350 family members of ICU patients in Turkey, 81.4% of respondents reported experiencing anxiety, while 94.2% reported symptoms of depression (Çelik et al., 2016). When PICS-F occurs, it can persist for 5–15 years, severely impacting patients’ and families’ quality of life, escalating medical resource needs, and burdening society (Gu et al., 2023; Voiriot et al., 2022). Research on the elevated incidence of PICS-F suggests that implementing appropriate intervention strategies is essential to prevent its onset and progression (Elliott et al., 2014). However, in recent years, the majority of studies pertinent to the field of critical care have predominantly concentrated on patients, often neglecting the experiences and needs of their families. In fact, regardless of whether ICU patients survive or not, their family members may develop post-ICU syndrome, and the psychological disorders of family members are more severe and last longer than those of ICU survivors (Fumis et al., 2015). This phenomenon may be attributed to the challenges faced by patients discharged from the ICU, which include physical and psychological impairments, diminished quality of life, and difficulties in reintegrating into society (Ho and Li, 2023). Approximately 25% of these patients require ongoing informal care services within 6 months to 1 year post-discharge, with around 80% of this care being provided by family members (Griffiths et al., 2013). As the main caregivers of discharged patients from the ICU, family members often have to undertake multiple responsibilities, including: being responsible for the patients’ diet, medication, physical care and medical arrangements, as well as independently undertaking all household chores, taking care of children, social life and other responsibilities that should have been borne by the patients (Choi et al., 2018; Czerwonka et al., 2015; van Beusekom et al., 2016). Some family members may reduce their working hours or leave their jobs to provide care for patients, resulting in a decline in their economic status and imposing a significant financial burden on the family (Zeng, 2023). Current evidence suggests that the stress experienced by family members during a patient’s hospitalization in the ICU elevates the risk of developing PICS-F (Davidson et al., 2017). However, there remains a paucity of effective intervention strategies to mitigate this stress.

In light of this, we established a multidisciplinary team to develop a psychological intervention termed Sensation Awareness Focused Training (SAF-T). Grounded in psychophysiological theory, SAF-T integrates Eye Movement (EM) techniques—derived from Eye Movement Desensitization and Reprocessing (EMDR) therapy—with breathing and relaxation exercises. By engaging working memory through dual-task loading, enhancing interhemispheric communication, performing smooth-pursuit eye movements, and practicing slow, deep breathing, this approach reduces sympathetic nervous system arousal. This process facilitates a calming response, interrupts negative thoughts, emotions, and behaviors, alleviates stress, and helps prevent the onset of anxiety and depression (Lee and Cuijpers, 2013). Eye movements not only activate large-scale default mode network activity while attenuating frontoparietal control network activity—thereby releasing functions such as self-reflection, memory retrieval, and associative learning while temporarily suppressing physiological arousal and the high cognitive load induced by traumatic memories—but may also activate two emotion regulation pathways: the cerebellar-thalamic-prefrontal pathway and the cerebellar-hypothalamic-limbic pathway (Zou et al., 2025). This dual activation minimizes defensive emotional responses during SAF-T. Voogd et al. found that eye movements can promote interhemispheric and intrahemispheric interactions, thereby facilitating adaptive memory integration (Voogd and Linda, 2017). Extensive literature also indicates that repeated eye movements may activate the parasympathetic nervous system and elicit a bodily relaxation response (Barrowcliff et al., 2003; Elofsson et al., 2008; Stickgold, 2002). Finally, the physiological effects of deep breathing in SAF-T, which activates the parasympathetic nervous system, contribute to stress and anxiety reduction (Jerath et al., 2006).

This study will employ a randomized controlled trial (RCT) design to examine the efficacy of SAT-F, a stress management intervention, in preventing PICS-F (stress, anxiety, depression, PTSD, and sleep quality) among family members of patients in the intensive care unit during their hospitalization.

### 1.1 Study aims and hypotheses

The primary objective of this RCT is to evaluate the immediate efficacy of the SAF-T intervention in alleviating psychological distress (a composite of stress, anxiety, and depressive symptoms) among family members of ICU patients, as measured on Day 3 post-enrollment (immediately after intervention completion). Secondary objectives include: (1) To assess the longitudinal trajectories of stress, anxiety, and depressive symptoms in family members receiving the SAF-T intervention, examining its sustained effects at Day 3 post-enrollment (post-intervention), upon patient transfer from the ICU, and at the Day 30 and Day 90 follow-ups. (2) To explore the potential of the SAF-T intervention for preventing PTSD symptoms, measured at Day 3 post-enrollment (post-intervention), upon patient transfer from the ICU, and at the Day 30 and Day 90 follow-ups. (3) To explore the effect of the SAF-T intervention on sleep quality, measured at Day 3 post-enrollment (post-intervention), upon patient transfer from the ICU, and at the Day 30 and Day 30 follow-ups.

Based on the objectives above, the following hypotheses are proposed: (1) Primary Hypothesis: Compared to family members receiving usual care (control group), those receiving the SAF-T intervention will demonstrate a significantly lower composite score of psychological distress after completing the 3-day intervention period (Day 3 post-enrollment). (2) Secondary Hypotheses: (1) Compared to the control group, the intervention group will show greater reductions or faster improvement in individual scores on the perceived stress scale (PSS-10), generalized anxiety disorder scale (GAD-7), and patient health questionnaire (PHQ-9) from baseline to each follow-up assessment. (2) Compared to the control group, the intervention group will have significantly lower impact of event scale-revised (IES-R) scores (indicating fewer PTSD symptoms). (3) Compared to the control group, the intervention group will have significantly lower sleep quality index (PSQI) scores.

## 2 Methods and analysis

### 2.1 Study design and setting

This research will employ a single-center, prospective, parallel-design, RCT protocol, meticulously following the CONSORT guidelines. This study will be conducted in the ICU of a tertiary-grade a general hospital located in China. The study protocol was approved by the ethics committee and registered in the Chinese Clinical Trial Registry.

#### 2.1.1 Participants

This study will recruit family members of ICU inpatients who fulfill the specified inclusion and exclusion criteria as research subjects. Members of the research team will review the electronic medical records within the hospital information system to preliminarily identify eligible family members. Subsequently, face-to-face communication and assessments will be conducted with those family members who meet the research criteria, ultimately leading to the selection of individuals who satisfy the study’s conditions. All participants will provide informed consent prior to their involvement in the study.

The inclusion criteria for this study are as follows: patients must be admitted to the ICU for a minimum duration of 24 h; family members, defined as parents, children, spouses, or siblings, must be aged 18 years or older; each patient will be represented by a single primary caregiver responsible for medical decision-making; family members must have no prior history of mental illness or severe organic diseases; and family members must provide informed consent and voluntarily agree to participate in the study. The exclusion criteria include: patients who are unidentified, unaccompanied, and lack financial support, which renders family contact unfeasible; and family members who possess severe hearing or speech impairments that hinder their ability to participate in the study. The withdrawal criteria for this study are as follows: incomplete assessment of baseline data and primary indicator evaluation data; and noncompliance with the research protocol as stipulated. When a participant withdraws from the study, researchers should make every effort to contact the individual through various methods, including phone calls and online communication, to inquire about and document the reasons for their withdrawal. All randomized participants will be analyzed according to the intention-to-treat (ITT) principle upon trial completion, irrespective of their adherence to or completion of the intervention protocol.

#### 2.1.2 Sample size

The sample size estimation for this study was calculated based on the results of a pilot study by Cairns (2018). This pilot study was selected as a reference for several reasons: both studies involve similar populations (family members of ICU patients), comparable intervention intensity (continuous intervention over 3 days, each lasting 15–20 min), similar assessment tools and outcome indicators (key indicators in this study were selected from those that performed well in the Cairns’ study, or alternative validated tools were chosen to address its limitations), and largely aligned assessment time points (the Cairns’ study assessed on Day 1, Day 3, Day 30, and Day 90; this study additionally included an assessment within 24 h after patient discharge to better reflect the interaction between patients and their families). In the pilot study (*N* = 10; 5 per group), the effect size for the outcome of perceived stress from baseline to 90-day follow-up was Cohen’s *d* = 1.13 (95% CI: −0.21 to 2.47), based on a mean difference of 4.4 points (intervention: 23.0 ± 2.30 vs. control: 25.0 ± 5.05). This effect size represented the most conservative estimate among all follow-up time points (Day 3: *d* = 1.50; Day 30: *d* = 1.23; Day 90: *d* = 1.13). Sample size estimation for the “independent sample *t*-test” was performed in G Power3.1 sample size calculation software,^1^ and the calculation formula is as follows: $n={\left[\frac{Z_{\alpha /2}+Z_{\beta }}{\delta /\sigma }\right]}^{2}(Q_{1}^{-1}+Q_{2}^{-1})$ (Faul et al., 2007; Faul et al., 2009). The parameter Settings are as follows: For the two-sided test, Cohen’s d is 1.13, Alpha is 0.05, the certainty Power (1-β) is 0.95, and the sample size is selected as N1 = N2. Finally, it was calculated that each group of the intervention study required 22 people. Considering a loss to follow-up rate of more than 20%, the minimum sample size included in each group was ultimately 28 people. When the sample size was sufficient, as many research subjects as possible were included in the study. Acknowledging the inherent variability and potential overestimation in pilot studies, we aimed for a conservative design by selecting a high statistical power to minimize the risk of Type *II* error.

To account for the inherent uncertainty in the pilot-derived effect size estimate, we conducted a sensitivity analysis using a range of more conservative effect sizes. The results are shown in Table 1. With 28 participants per group, our trial maintains 80% power to detect a medium-to-large effect (*d* = 0.7), which we consider a clinically meaningful target.

**Table 1:** Sensitivity analysis of sample size under different effect size scenarios.

Scenario	Effect size (d)	Power	Required N per group	Our *N* = 28 achieved power
Pilot estimate (Day 90)	1.13	0.95	20	>99%
Large effect	0.80	0.95	36	88%
Medium-large effect	0.70	0.95	46	80%
Medium effect	0.50	0.80	64	51%
Lower bound of 95% CI	0.21	0.80	358	12%

#### 2.1.3 Randomization and blinding

A comprehensive randomization procedure was implemented in which an independent third-party researcher utilized the Research Randomizer software^2^ to generate a random sequence of numbers ranging from 1 to 56. Each number was then placed into a correspondingly numbered, sealed, opaque envelope. To maintain allocation concealment, all envelopes were securely stored by designated personnel. Following the identification of the research subjects, the researchers contacted the custodians of the random number envelopes via telephone to inquire about the grouping procedure. The custodians subsequently activated the envelopes in sequential order and organized the subjects accordingly. When the number contained within the envelope is odd, the research participant is allocated to the intervention group; conversely, when the number is even, the participant is assigned to the control group.

Due to the nature of the face-to-face psychological intervention, complete blinding was not feasible for the intervention providers or research subjects. To minimize potential bias, the following blinding procedures will be implemented: (1) Blinding of outcome assessors: Staff responsible for outcome assessment will be kept unaware of group allocation and will have no involvement in the intervention or randomization. (2) Blinding of data analysts: Analysts will remain blinded to group assignment until the final analysis is completed and the database is locked. (3) Procedures to maintain blinding: To safeguard blinding, all study materials will use coded participant IDs without any group indication. The allocation list will be kept by an independent research coordinator not involved in assessment or analysis.

### 2.2 Intervention

#### 2.2.1 Control group

Participants randomized to the control group will receive standard care, which is strictly limited to informational and procedural support. The components are as follows: (1) During ICU hospitalization. (1) Informational support: Nursing staff will provide a concise introduction to the ICU environment, equipment, and visiting policies. (2) Medical staff will communicate essential information regarding the patient’s clinical status, treatment plan, and progress. (3) Basic care guidance: Family members will be instructed on fundamental, task-oriented nursing skills necessary for patient care (e.g., assisting with rehabilitation exercises, basic daily care) as appropriate. (4) Psychological support: To minimize contamination, control group participants will not receive any proactive, structured, or protocol-driven psychological counseling from the ICU clinical team or study personnel during the 3-day intervention window. This includes, but is not limited to: stress management techniques, relaxation training, cognitive reframing, or any form of psychotherapy. If a family member exhibits signs of significant psychological distress that require clinical attention, the ICU clinical team will follow the hospital’s standard protocol, which typically involves notifying the attending physician and/or initiating a referral to the hospital’s psychiatric liaison service. Such instances will be documented as exceptional clinical events and are not considered routine components of standard care. (2) Post-discharge follow-up. The health guidance and follow-up mentioned will be confined to routine medical advice (e.g., medication management, wound care) and the administration of outcome assessments for this study. It will not include systematic psychological support or counseling components. (3) Documentation of co-interventions. To ensure transparency and enable appropriate statistical adjustment, any instance of substantial psychological support provided to a control group participant outside the study protocol will be systematically documented by the research team using a standardized co-intervention documentation form. The following information will be recorded: type of support, duration, timing, reason/trigger. These data will be monitored throughout the trial by the research team. In the primary analysis, between-group comparisons will be conducted regardless of co-intervention exposure. As a pre-specified sensitivity analysis, we will compare the primary outcomes after excluding participants who received any protocol-prohibited psychological support, to assess the potential impact of such co-interventions on the study results. Additionally, if the frequency of co-interventions differs substantially between groups, this will be addressed in the interpretation of findings.

#### 2.2.2 Experimental group

The experimental group will receive the SAF-T intervention in addition to the standard care provided to the control group. The SAF-T intervention was developed by the research team following an extensive review of the literature. The team comprises ICU clinicians, specialized ICU nurses, and psychiatrists and psychologists, all of whom possess substantial clinical expertise. The detailed SAF-T intervention script is provided in Table 2. Before the commencement of the formal study, to ensure the standardized implementation of the intervention, a clinical psychotherapist from the intervention development team provided SAF-T training to another researcher in the study team with EMDR learning experience. The training covered the theoretical framework and specific procedures of SAF-T, as well as the instruction of relevant psychological intervention techniques. The training process is monitored by all members of the intervention-constructed team. To ensure the consistency of the SAF-T intervention protocol, the psychological intervention will be delivered by a fixed, dedicated research member. Following each session, the interventionist will complete a session checklist to document adherence to the specific operational procedures. To proactively monitor participant well-being, the interventionist will conduct structured inquiries at the beginning and end of each session using a combination of open-ended and closed-ended questions. Throughout the session, the interventionist will remain vigilant for signs of emotional deterioration, such as crying, agitation, reduced verbal output, or expressions of hopelessness. Should such signs emerge and significantly impede the session’s progress, the intervention will be paused temporarily. The interventionist will then provide support, ensure the participant’s immediate stability, and respectfully inquire about their willingness to continue. If resumption is not feasible, the session will be rescheduled. Furthermore, participants are explicitly informed that they may pause or withdraw from the intervention at any time due to emotional discomfort without any penalty or consequence.

**Table 2:** SAF-T intervention script.

Component	Description
Objective	To alleviate stress, anxiety, and depression in ICU patients’ family members via SAF-T, empowering them for independent stress management. Skill application is encouraged based on personal relevance, not mandated.
Participants	Eligible family members of ICU patients meeting inclusion/exclusion criteria.
Duration	15–20 min/session for 3 consecutive days.
Location	Quiet, comfortable conversation room.
Intervention process	1. Pre-intervention: Schedule session; obtain informed consent; collect baseline data. 2. Intervention Sessions: ① Introduction/Review: Explain purpose and build trust/Review the previous homework and practical work situation. (About 3 min) ② Stress source identification: Link stress responses to ICU-related factors. (About 3 min) ③ SAF-T: Sit facing the subject, place your hand within their line of sight, and perform rapid parallel sliding for 1 min. Moving left to right counts as once, and right to left as twice, repeating for at least 40 cycles per minute. Keep arm swing within 90°. The subject should keep their head still, follow the researcher’s finger movement with their eyes only, and maintain slow, rhythmic deep breathing. The detailed SAF-T intervention procedures can be found in supplement 1. (About 10 min) ④ After the SAF-T: Collect participant feedback and complete the session checklist for this case. (About 4 min) ⑤ Practice & Generalization: If the participant is willing, sessions on Days 2 and 3 will focus on independent practice; discuss real-life application after final session. 3. Follow-up: Assessments at 3, 30, 90 days post-intervention and within 24 h after ICU discharge via outpatient/phone/online.

### 2.3 Outcomes

#### 2.3.1 Primary outcome and endpoint

The primary outcome of this trial is the psychological distress level of family members at the primary endpoint, which is defined as Day 3 (immediately after the completion of the SAF-T intervention).

#### 2.3.2 Secondary outcomes

The secondary outcomes of this study focus on the trajectories of individual symptoms. To investigate the sustained and evolving effects of the intervention, individual scores for stress, anxiety, depression, PTSD, and sleep quality will be analyzed across a complete longitudinal time series. This series includes assessments at baseline (Day 1), immediately post-intervention (Day 3), within 24 h after the patient’s discharge from the ICU, and at the 30-day and 90-day follow-ups.

### 2.4 Measuring tools

(1) This study will employ the PSS-10 to assess the stress levels of family members of patients in the ICU, as previous research has demonstrated that the PSS-10 exhibits superior reliability and structural validity in practical applications within China (Huang et al., 2020).
(2) GAD-7 will be utilized to evaluate the anxiety levels among family members (Spitzer et al., 2006).
(3) PHQ-9 will be utilized to evaluate the depression levels among family members (Williams et al., 2005).
(4) PTSD Symptoms: The impact of IES-R questionnaire will be utilized to assess the severity of PTSD among family members (Horowitz et al., 1979).
(5) Sleep quality: The Pittsburgh PSQI will be utilized to assess the sleep quality of family members at all-time points (Buysse et al., 1989).

### 2.5 Data collection

We will use the general information questionnaire to collect demographic characteristics of patients and their families. The general information questionnaire was designed by the researchers themselves based on extensive literature reading, which included pertinent data related to ICU patients and their families, and consisted of a total of 17 items.

To address information bias arising from inter-collector variability, standardized paper-based questionnaires will be administered by data collectors after first obtaining informed consent from family members. Baseline data will first be gathered on-site. Subsequently, follow-up data collection will be carried out by pre-designated data collectors at predetermined intervals following the intervention. Data collection will primarily be conducted via telephone interviews. It will occur at pre-specified time points: on the Day 1 (pre-intervention), on the Day 3 (post-intervention), within 24 h post-discharge, as well as on the Day 30 and Day 90. Additionally, ICU discharge follow-up data will be gathered within 48 h on both the 30th and 90th study days (Table 3).

**Table 3:** Key variables, measures, and data collection time points.

Outcomes	Measures	Data collection time points				
		Study Day 1	Study Day 3	≤24 h post-ICU discharge	Study Day 30	Study Day 90
Anxiety	GAD-7	**√**	**√**	**√**	**√**	**√**
Depression	PHQ-9	**√**	**√**	**√**	**√**	**√**
PTSD	IES-R	**√**	**√**	**√**	**√**	**√**
Pressure	PSS-10	**√**	**√**	**√**	**√**	**√**
Sleep quality	PSQI	**√**	**√**	**√**	**√**	**√**
Demographic characteristics	General information questionnaire	**√**				

### 2.6 Statistical analysis

In this study, EpiData3.1 software will be utilized for data entry, while IBM SPSS Statistics version 27.0 will be employed for data analysis.

All statistical tests were two-sided with *α* = 0.05, and *p* < 0.05 indicated significance. Categorical variables, including family member demographics, will be presented as counts and percentages and compared between groups using the *chi-square* test or *Fisher’s* exact test, as appropriate. Continuous variables will be assessed for normality using the *Shapiro–Wilk* test. Normally distributed data will be summarized as mean ± standard deviation, while non-normally distributed data will be summarized as median and interquartile range. For baseline comparisons of continuous variables, independent samples *t*-tests or *Mann–Whitney U* tests will be applied accordingly. For the intention-to-treat (ITT) analysis, missing data from participants who withdraw during the study will be handled using multiple imputation (MI). Specifically, the multiple imputation module in SPSS version 27.0 will be employed to create five complete datasets using the fully conditional specification method, with group allocation, time point, baseline scores, and demographic characteristics included as predictor variables. The imputed results will then be pooled for analysis.

Primary Outcome Analysis: The primary analysis will follow the ITT principle, including all randomized participants. The efficacy of the SAF-T intervention will be assessed by comparing the psychological distress composite score at the primary endpoint (Day 3) between the intervention and control groups, adjusting for the baseline composite score. (1) Calculation of the composite score. To ensure comparability across time points, all composite scores will be standardized using the means and standard deviations calculated from the Day 3 assessments. For each participant at Day 3, the composite score will be calculated as follows: First, for each of the three scales (PSS-10, GAD-7, and PHQ-9) administered at Day 3, the mean ( ${\overset{ˉ}{x}}_{k}$ ) and standard deviation ( $SD_{k}$ ) will be calculated across all participants, where k=1,2,3 represents the three scales, respectively. Second, for each participant i , the raw score on each scale ( $x_{\mathrm{ik}}$ ) will be standardized to a Z-score: $Z_{\mathrm{ik}}=\frac{x_{\mathrm{ik}}-{\overset{-}{x}}_{k}}{SD_{k}}$ . Finally, the three standardized scores will be summed to obtain the participant’s psychological distress composite score: $\text{CompositeScor}e_{i}=Z_{i,\mathrm{PSS}-10}+Z_{i,\mathrm{GAD}-7}+Z_{i,\mathrm{PHQ}-9}.$ (2) Primary statistical model. The primary between-group comparison at Day 3 will be performed using an analysis of covariance (ANCOVA), with the Day 3 composite score as the dependent variable, experimental group as the fixed factor, and the baseline composite score as a covariate. Prior to ANCOVA, normality of residuals and homogeneity of regression slopes will be assessed. If the assumptions of ANCOVA are violated, a non-parametric alternative (e.g., Quade’s rank ANCOVA) will be applied. (3) Effect size and validation. To assess the clinical significance of the intervention effect, Cohen’s d effect size will be calculated, with *d* = 0.2 considered a small effect, *d* = 0.5 a medium effect, and *d* = 0.8 a large effect (Bartko et al., 1988). As a validation step, the theoretical standard deviation of the composite score will be computed using the variance sum theorem: $SD_{\text{theoretical}}=\sqrt{3+2(r_{12}+r_{13}+r_{23})}$ . where $r_{12}$ , $r_{13}$ , and $r_{23}$ represent the Pearson correlation coefficients between each pair of the three scales. This theoretical value will be compared with the observed standard deviation to confirm the construct validity of the composite score. (4) Sensitivity analyses. As a sensitivity analysis, a per-protocol (PP) analysis will be conducted to evaluate the robustness of the primary ITT results. Additionally, to assess the potential impact of contamination, we will perform an analysis excluding control group participants who received protocol-prohibited psychological support during the intervention window.

Analysis of Secondary and Longitudinal Outcomes: For longitudinal analyses of secondary outcomes—including the individual trajectories of PSS-10, GAD-7, and PHQ-9 scores, as well as PSQI and IES-R scores across time points (Day 3, discharge, Day 30, Day 90)—linear mixed-effects models (LMMs) will be employed. This approach handles missing data under the missing-at-random (MAR) assumption and accounts for within-subject correlation. Separate LMMs will be fitted for each continuous secondary outcome. Each model will include: (1) Fixed Effects: Treatment group, time (as a categorical factor), the group × time interaction, and the corresponding baseline score of the outcome as a covariate. (2) Random Effects: A random intercept for each participant will be included to account for individual variability. (3) Covariance Structure: An unstructured covariance matrix will be initially specified to model the within-subject errors, with the final structure chosen based on model fit indices (e.g., Akaike Information Criterion). The group × time interaction will be the effect of primary interest, testing whether the change in the outcome over time differs between groups. If the interaction is significant, simple effects analyses (e.g., between-group comparisons at each time point) will be conducted. For the primary ITT analysis, missing outcome data at Day 3 will be handled using the Multiple Imputation (MI) method. Results from analyses on each dataset will be pooled using Rubin’s rules. For longitudinal LMMs, the full-information maximum likelihood (FIML) estimation inherent to the method will be utilized.

Handling of Multiplicity: All analyses of secondary outcomes and longitudinal trajectories are considered exploratory. Results from these analyses will be presented with point estimates and 95% confidence intervals to indicate the magnitude and precision of effects. *p*-values from these exploratory analyses will be interpreted descriptively and with caution, without formal adjustment for multiple testing, to avoid inflating Type II error rates while generating hypotheses for future research.

Additional Analyses: Intra-group changes from baseline to specific time points for normally distributed data will be assessed using paired *t*-tests, or the *Wilcoxon* signed-rank test for non-normally distributed data. And we will assess the sensitivity of the results to potential contamination by conducting exclusion analysis and reclassification analysis on the control group participants who received additional support.

### 2.7 Ethical considerations

This study received approval from the Medical Ethics Committee, and informed consent was obtained from all research participants. The methods, aim, and potential risks associated with the study were thoroughly explained to the participants. Each participant will sign an informed consent form. Furthermore, all researchers are required to maintain strict confidentiality regarding the participants’ information.

## 3 Discussion

Family members of ICU patients frequently serve as primary caregivers and key decision-makers for critically ill individuals, yet they often experience substantial psychological stress manifesting as anxiety or depression. The stress can manifest in symptoms such as anxiety and depression, significantly elevating the risk of developing PICS-F. However, the majority of research conducted on intensive care units predominantly emphasizes patient experiences, often neglecting the significant emotional distress faced by family members. Research indicates that alleviating stress experienced by the families of patients during their stay in the ICU can significantly reduce the incidence of PICS-F (Davidson et al., 2017). The majority of existing intervention studies specifically addressing PICS-F predominantly focus on communication strategies or educational frameworks within the ICU (Rahimi-Bashar et al., 2025). Communication frequently focuses on conveying information to the patient’s family members regarding the current status of the patient’s illness, treatment strategies, and progress. However, it often neglects the provision of psychological and emotional support to the family members. Herein, we present a comprehensive stress management intervention developed by our research team. This study protocol describes a RCT to evaluate the efficacy of the SAF-T intervention in reducing psychological distress among ICU patients’ family members. From a clinical perspective, the brief, manualized SAF-T protocol is designed for potential integration into ICU workflows, offering a scalable model for early psychosocial support that may mitigate the risk of PICS-F. On a methodological level, this trial aims to generate high-quality evidence through a rigorous design. Regarding policy and practice, demonstrating the effectiveness of this low-cost, preventive intervention could inform clinical guidelines and support the implementation of family-centered care in critical care settings.

### 3.1 Limitations

The potential limitations of this study encompass the risk of attrition during the 90-day follow-up period. Specifically, post-discharge from the hospital, the collection of data may become increasingly challenging. Furthermore, the single-center design of this study, constrained by limited financial and human resources, will restrict the generalizability of the findings.

## 4 Conclusion

This protocol outlines the rationale and design for a RCT evaluating the efficacy of the SAF-T intervention—a brief, structured psychological program—in preventing PICS-F among family members of critically ill ICU patients. By employing a rigorous methodological framework that includes an active control group, longitudinal assessment, and comprehensive plans for fidelity monitoring and safety management, the study aims to generate high-quality evidence on the potential of early, scalable psychosocial support. If proven effective, the SAF-T intervention could provide a feasible model for integration into routine critical care practice, ultimately contributing to improved family well-being and the advancement of family-centered care.

## Footnotes

1
www.gpower.hhu.de

2
randomizer.org
