# Supplementary material for: Evaluating a stress management intervention effects on post-ICU syndrome in families: study protocol for a single-center, prospective, parallel-design, randomized controlled trial
Source: Front Psychol. 2026 Apr 13;17:1783279. doi: 10.3389/fpsyg.2026.1783279 (PMC13112261; doi:10.3389/fpsyg.2026.1783279)
Supplement: Supplementary file 1 [file Data_Sheet_1.docx]

**Sensation Awareness Focused Training (SAF-T) Intervention Procedure**

Inform the visitors that this exercise is designed to alleviate or potentially eliminate physical discomfort. It is particularly beneficial in addressing uncomfortable emotions such as anxiety, depression, and anger. Should the participants achieve a high level of proficiency in this technique, they will be able to integrate it into their daily routines for effective self-management of stress. Furthermore, if the participants find this technology to be effective, they may also share it with their family and friends.

**Procedure**

1. **Introduction**
   - We will now begin an exercise where there are no right or wrong responses.
   - Now... Please think of a memory that troubles you and focus your attention on the physical sensations that accompany this distress.
   - When you think about this memory, rate it on a scale of 0 to 10. A score of 0 means you don't feel any disturbance, and a score of 10 means the highest disturbance you know. How many points do you feel now?
2. **Breathing Guidance**
   - Ok...... Watch your breathing now...... Inhale the air and let it enter your abdomen through your nose, while counting for 4 seconds (1... 2... 3... 4) ... Then, hold it gently for 2 seconds (1... 2), then exhale air and count for 4 seconds at the same time (1... 2... 3... 4) ... Then continue to take three deeper and slower breaths... Just like this...
   - Repeat 3 cycles of deeper, slower breaths.
3. **Visual Tracking**
   - Let's maintain the current way of breathing... Pay attention to my fingers... Keep your head still and only follow my fingers with your eyes... (Move the fingers for at least one minute) ...
4. **Post-Exercise Reflection**
   - Now, let's spend one minute taking six deeper and slower breaths, just like just now... And then come back to yourself... Go to your own center.
   - How do you feel now?...
5. **Reinforcement & Home Practice**
   - If this **Sensation Awareness Focused Training (SAF-T)** proves helpful, practice it daily in a comfortable environment until internalized.
   - Apply this technique when experiencing anxiety, depression, overwhelming stress, or somatic pain.
   - Now… let's return to this room with total awareness and feel your feet on the ground...
